# Investigation of Annealing Temperature Effect of Tin Oxide on the Efficiency of Planar Structure Perovskite Solar Cells

**DOI:** 10.3390/nano15110807

**Published:** 2025-05-28

**Authors:** Ahmed Hayali, Maan M. Alkaisi

**Affiliations:** 1 Department of Computer Networks and the Internet, College of Information Technology, Ninevah University, Mosul 41001, Iraq; 2 Department of Electrical and Computer Engineering, University of Canterbury, Christchurch 8041, New Zealand; 3 The MacDiarmid Institute for Advanced Materials and Nanotechnology, Wellington 6140, New Zealand

**Keywords:** perovskite solar cells, low temperature, tin oxide, electron transport layer, spin coating

## Abstract

Tin oxide (SnO_2_) is an attractive candidate for the electron transport layer (ETL) in perovskite-based solar cells because of its low temperature process requirement. The ability to form ETL layers at low temperatures opens up opportunities for the use of flexible and low-cost materials suitable for photovoltaic applications. The ETL is necessary for the extraction of electrons and charge separation from the perovskite active layer. Herein, we present a study of the effect of annealing temperature on SnO_2_ used as an ETL. The annealing temperature of the SnO_2_ has a considerable effect on the morphology, crystallinity, grain size, and surface topography of the SnO_2_ layer. The surface properties of the ETL influence the structural properties of the perovskite films. In this study, the annealing temperature of the SnO_2_, deposited using spin coating, was changed from 90 °C to 150 °C. The SnO_2_ films annealed at 120 °C resulted in reduced surface defects, improved electron extraction, and produced a significant increase in the grain size of the perovskite active layers. The increase in grain size led to improved efficiency of the PSCs. Devices annealed at 120 °C yielded PSCs with an average efficiency of 15% for a 0.36 cm^2^ active area, while devices treated at 90 °C and 150 °C produced an average efficiency of 12%. The PSCs fabricated at low temperatures provide an effective technique for low-cost manufacturing, especially on flexible and polymer-based substrates.

## 1. Introduction

PSCs have been explored as a promising new generation of photovoltaic devices due to their outstanding light-harvesting properties, low fabrication cost, and diverse range of applications. Particularly, the power conversion efficiency (PCE) of PSCs has seen sharp increases over the past 15 years. In 2009, the efficiency of the first perovskite device prepared by Kojima et al. was 3.8% using a 0.2 cm^2^ active area [1]. Then, in just one decade of extensive efforts, the efficiency of PSCs has reached over 26% [2]. Due to the fact that tin oxide (SnO_2_) possesses good optical and electrical properties, SnO_2_ has been extensively utilized as an ETL in the fabrication of PSCs. Generally, the SnO_2_ can be deposited by different methods such as magnetron sputtering [3], chemical vapor deposition (CVD) [4], atomic layer deposition (ALD) [5], the hydrothermal process [6], electrodeposition [7], nebulized spray pyrolysis [8], and the sol-gel process [9]. In this work, the SnO_2_ ETL was deposited using a low-cost spin coating process.

SnO_2_ has been studied as a possible alternative material to titanium dioxide (TiO_2_) for ETLs. This is due to its high electron mobility suitable for electron extraction and similar physical structural properties to TiO_2_ [10]. Generally, TiO_2_ has been widely utilized as an ETL for PSCs [11,12,13,14,15]. However, TiO_2_ has low electron mobility and requires a high annealing temperature up to 500 °C for a duration of one hour [16,17,18]. The high temperature increases the device manufacturing cost and limits the use of flexible substrates. SnO_2_ has been explored as a substitute for TiO_2_ and ZnO to reduce manufacturing costs and improve the efficiency of PSC devices [19,20]. Guangfeng et al. have described the fabrication of PSCs using sputtered SnO_2_ as an ETL and achieved an efficiency of 18.2% using a small active area of 0.14 cm^2^ [21]. In 2015, Weijun et al. fabricated a perovskite film with CH_3_NH_3_PbI_3_ composition and utilized nanocrystalline SnO_2_ as an ETL to achieve a PCE of 16% [22]. In the same year, Weijun et al. prepared a vacuum-processed CH_3_NH_3_PbI_3_ perovskite composition and used low-temperature SnO_2_ as an ETL that achieved an average efficiency of 14% [23]. Qi et al. prepared perovskite using solution-processed SnO_2_ nanoparticles as an ETL and achieved an efficiency of 19.9% using an active area of 0.108 cm^2^ [24].

Generally, the photovoltaic devices and their photoelectric properties are significantly influenced by the crystallographic orientation of the perovskite active layer crystal structure [25]. The interfaces between the perovskite active layer and ETL show high defect concentrations that influence the perovskite film morphology. This causes a drop in open circuit voltage. Therefore, the topography and grain size of perovskite films are affected by the surface roughness of ETL, which influences the performance of devices [26].

The mesoscopic perovskite structure conventionally uses TiO_2_ as the ETL material; however, TiO_2_ needs to be annealed at a high temperature of 500 °C, which may hinder the large-scale commercialization of perovskite devices. Low temperatures and a simple fabrication method are the two parameters that are essential requirements for researchers and for the photovoltaic industry [27,28,29].

In this study, a low annealing temperature of SnO_2_ was examined in the range from 90 °C to 150 °C. The triple cation perovskite with n-i-p planar structure and a chemical formula of“CsI_0.05_[(FAPbI_3_)_0.85_ (MAPbBr_3_)_0.15_]_0.95”_was prepared at room temperature, as mentioned elsewhere [11,13]. Generally, the interfaces between the perovskite film and the ETL play a significant role in the performance of the PSCs. We have investigated the influence of changing the annealing temperature on the properties of SnO_2_ and its impact on the performance of PSCs. Herein, to understand the effect of changing the annealing temperature of SnO_2_ on the efficiency of PSCs, the optical and electrical properties of SnO_2_ and the perovskite active layer deposited on the ETL with different annealing temperatures were studied.

Our results suggest that perovskite films grown outside the glovebox and under ambient laboratory conditions (25 °C temperature and 45% humidity) are suitable for flexible substrate applications when combined with a low-temperature SnO_2_ ETL process.

## 2. Materials and Methods

### 2.1. Materials

All materials in this study were used as received from suppliers without any purification. The substrates used were “fluorine-doped tin oxide (FTO) soda lime glass with resistivity (12–15 Ω/sq), purchased from MSE pro^TM^ supplies (Tucson, AZ, USA). Methylammonium bromide (MABr) and formamidinium iodide (FAI) were purchased from GreatCell Solar. Lead (II) iodide (PbI_2_) 99.9% and lead (II) bromide (PbBr_2_) 99.9% were bought from Luminescence Technology Corp. (Lumtec), Hsin-Chu City, Taiwan. Acetonitrile anhydrous (99.8%) tin(II) chloride dehydrate SnCl_2_·2H_2_O, spiro-MeOTAD 99% (HPLC), chlorobenzene (CBZ) (95%), and 4-tert-Butylpyridine (TBP 98%) were procured from Sigma-Aldrich (Saint Louis, MO, USA). Cesium iodide (CsI 99.998%) was acquired from Alfa Aesar (Leicestershire, UK). Dimethyl sulfoxide (DMSO, 99.9%) and *N,N*-dimethylformamide (DMF, 99.5%) were bought from Fisher Scientific (Waltham, MA, USA).”

### 2.2. Methods

In this research, the “FTO glass substrate (12–15 Ω/sq)” was etched from one edge using zinc powder and dilute HCL to avoid a short-circuit problem. Then, the FTO glass substrate was washed with deionized water, followed by cleaning with acetone, methanol, and IPA each for 15 min in an ultrasonic bath. The ETL consists of two main layers: a compact TiO_2_ layer and an SnO_2_ layer. The compact TiO_2_ was prepared using a DC-sputtering technique with a titanium target and argon (6 sccm)-to-oxygen (12 sccm) ratio as mentioned elsewhere in more detail [11]. To clean and improve the hydrophilicity of the compact TiO_2_ surface, oxygen plasma ashing was applied for 10 min before SnO_2_ deposition. The SnO_2_ was prepared by dissolving 22.565 mg of SnCl_2_·2H_2_O in 1 mL of ethanol. Then, the SnCl_2_·2H_2_O precursor solutions were applied on the compact TiO_2_ by spin coating at 3000 RPM for 40 s. Then, it was heated on a hot plate in a laboratory at 120 °C for 30 min. In this study, the annealing temperature of SnO_2_ was investigated at three temperatures: 90 °C, 120 °C, and 150 °C.

**Figure 1:**
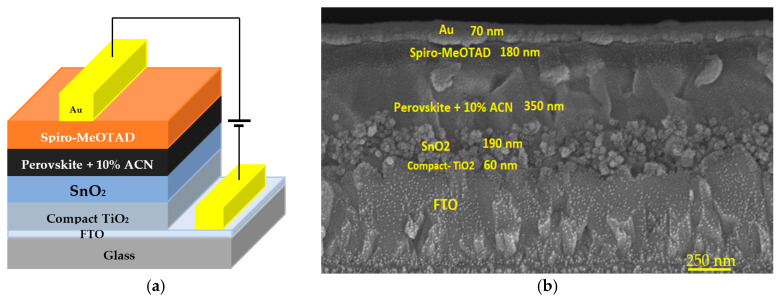
An architecture of the perovskite device (**a**) and an SEM image of the cross-sections of the perovskite device (**b**).

The triple cation perovskite was prepared by two methods, as explained in more detail elsewhere [11,12,13]. The main material compositions used to prepare the perovskite material were MABr (0.2M), FAI (1M), PbBr_2_ (0.2M), PbI_2_ (1.1M), and CsI (1.5M), and then a 10% solution of acetonitrile was added to the perovskite precursor solution. The resulting perovskite composition was applied on the ETL in an ambient air laboratory using two spin coating steps. First, 100 µL of precursor solution was spun onto the SnO_2_ at 1000 RPM for 15 s. This coated the entire sample. Second, the spinning speed was increased to 6000 RPM for 30 s. Finally, after 20 s waiting time, 200 µL of CBZ was dispensed on the samples before the end of the spinning to dispose of the DMSO/DMF. Then, the perovskite film was annealed on the hot plate at 100 °C for half an hour, as mentioned elsewhere [13]. The hole transport layer (HTL) was prepared by dissolving spiro-MeOTAD (80 mg) in chlorobenzene (1 mL) and adding 29 μL of 4-tert-butylpyridine (TBP), before 18ΜL of Li^+^ salt TFSI was added. A total of 100 µL of this composition was dispensed on the active layer and spun at 4000 RPM for 20 s. Finally, the gold electrodes of the device were prepared by electron beam evaporation. The architecture of the full perovskite device and layer thicknesses utilized in this study is shown in Figure 1.

The PCE of the perovskite device was measured using a sunlight simulator AM 1.5G ABET Sun3000 (100 mW/cm^2^), Milford, USA, and a Keithley meter. A Cary 6000i UV spectrometer (Waldbronn, Germany) was utilized to measure the transparency and absorption spectrum of the SnO_2_ prepared with different annealing temperatures. The grain size and topography of the SnO_2_ and perovskite film deposited on the SnO_2_ treated with different temperatures were measured using a Scanning Electron Microscopy tool (Raith-150) (Dortmund, Germany). A Rigaku Smart-Lab X-ray system (Tokyo, Japan) was used to measure the XRD spectra of the perovskite film deposited on the SnO_2_ film with different annealing temperatures. The morphology and surface roughness of SnO_2_ with different annealing temperatures were measured using Digital Instruments AFM from Veeco Instruments Inc. (Aschheim/Dornach, Germany). All measurements of PSCs were conducted in the ambient laboratory at 25 °C temperature and 45% humidity.

## 3. Results

### 3.1. Structural and Optical Properties of the SnO_2_ and Perovskite Films

**Figure 2:**
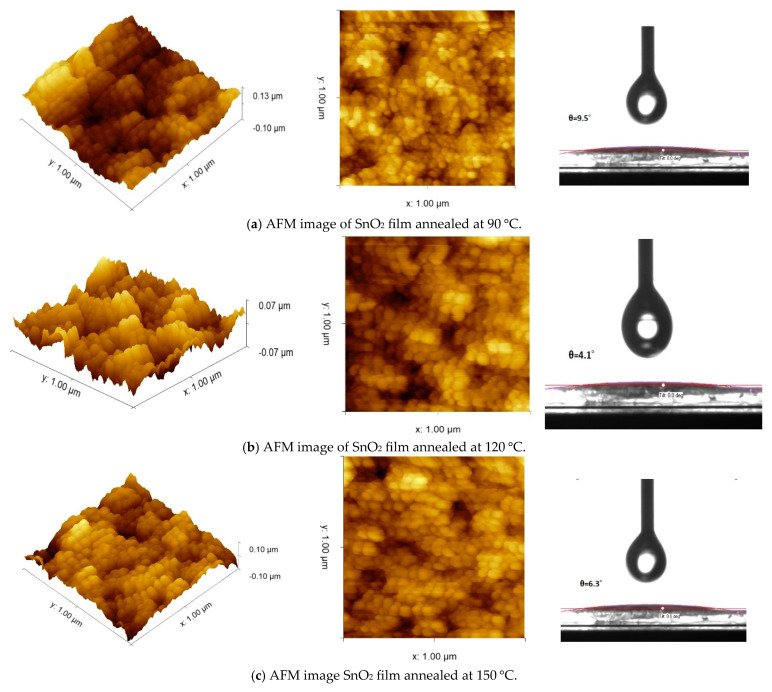
(**left**) 3D-AFM images, (**middle**), 2D-AFM images, and on the (**right**), contact angle measurement of SnO_2_ film annealed at (**a**) 90 °C, (**b**) 120 °C, and (**c**) 150 °C.

The interface properties between the C-TiO_2_ and SnO_2_ are critical for the electron extraction and recombination dynamics. C-TiO_2_ was deposited on FTO glass, and then SnO_2_ was deposited on C-TiO_2_ to decrease backflow of electrons [30]. The perovskite was deposited on the optimized ETL (SnO_2_/C-TiO_2_ to enhance the crystallinity of the perovskite active layer during the annealing method and to reduce the hysteresis of the perovskite devices [31]. The annealing temperature of SnO_2_ was changed from 90 °C to 150 °C. In this study, atomic force microscopy (AFM) was utilized to image the surface morphology of SnO_2_ annealed at different temperatures. Figure 2 shows the 3D-AFM and 2D-AFM images of SnO_2_ film annealed at 90 °C, 120 °C and 150 °C. The x–y axes measure from 0 to 1 um and the z-axis ranged from −0.1 µm to 0.13 µm. The root mean square (RMS) surface roughness value of the SnO_2_ films annealed at 120 °C is 14 nm, while the RMS values of the SnO_2_ annealed at 90 °C and 150 °C were 23 nm and 20 nm, respectively. This indicates that the SnO_2_ film annealed at 120 °C exhibits better homogeneity and produces a smoother surface. Moreover, it provides a more uniform surface and a denser grain structure, as shown in Figure 2. These SnO_2_ surface properties have led to a larger perovskite grain size [32]. The presence of dark and light areas in the AFM image in Figure 2 does not necessarily indicate the presence of cavities in the surface, but rather indicates differences in heights.

A contact angle (θ) measurement was used to measure the “surface wettability (hydrophilicity).” The surface energy properties of phase separation in the perovskite active layer are affected by the uniformity across the ETL materials. Measuring the contact angle θ provides an indication of the surface energy of each layer. The low θ of the ETL demonstrates a good matching between the ETL and the perovskite active layer, which enhances the perovskite film morphology properties [33]. It is shown in Figure 2 that the θ of SnO_2_ annealed at 120 °C is 4.1°, while the θ values of SnO_2_ treated at 90 °C and 150 °C are 9.5° and 6.3°, respectively. It is found that SnO_2_ heated at 120 °C achieved a low contact angle and high hydrophilicity, which can be attributed to the decrease in the surface roughness of the SnO_2_. Since SnO_2_ ETL acts as a seed layer for the perovskite deposition, the surface roughness and wettability of SnO_2_ will affect the topography and grain size of the perovskite active layer [15].

**Figure 3:**
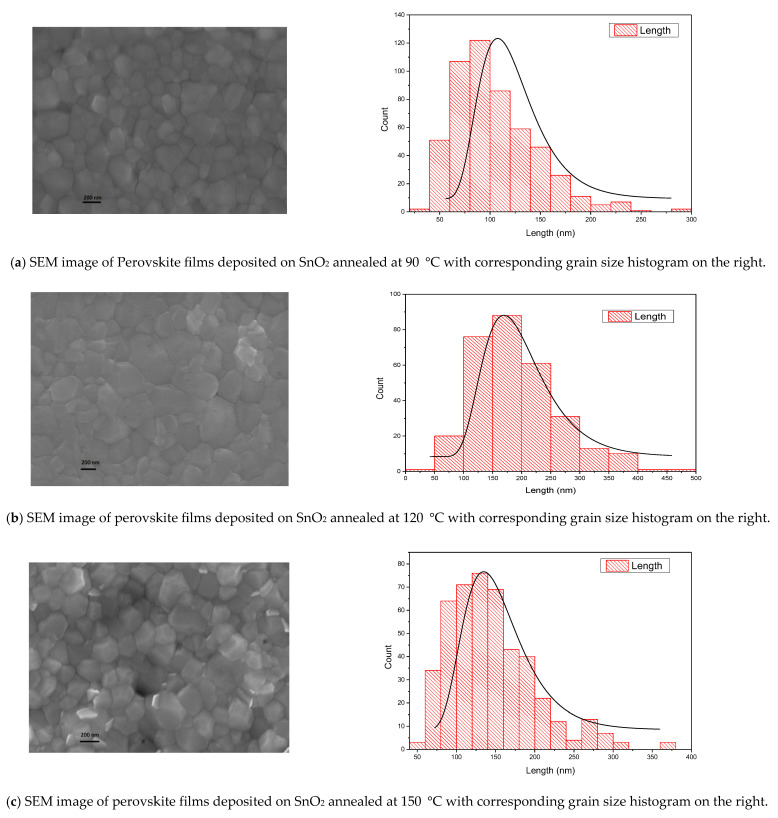
On the left SEM images of the surface morphology of the perovskite active layer deposited on SnO_2_ annealed at (**a**) 90 °C (**b**) 120 °C, (**c**) 150 °C. On the right is a histogram graph for each annealing temperature.

Image J and Origin 9 software (version 1.54p) were used to compute the average grain size and draw a histogram curve. Figure 3 displays the SEM images of the surface morphology of perovskite active layers deposited on SnO_2_ films annealed at 90 °C, 120 °C, and 150 °C. The following histogram displays the average grain size for each annealing temperature. It is found that the perovskite film deposited on SnO_2_ annealed at 120 °C showed a uniform structure with an average grain size of 180 nm, which is larger than the grain size of other perovskite films deposited on SnO_2_ annealed at 90 °C and 150 °C. The average grain sizes of perovskite film deposited on SnO_2_ annealed at 90 °C and 150 °C are 115 nm and 130 nm, respectively, as shown in the histogram of Figure 3. We found that perovskite films with a large grain size produce a low series resistance (R_s_), resulting in improved efficiency of the device [32]. Our results showed that the perovskite active layer deposited on SnO_2_ annealed at 120 °C has a larger grain size and fewer defects than other perovskite films deposited on SnO_2_ annealed at 90 °C and 150 °C. Larger grain size leads to enhancement in carrier conduction properties [34].

**Figure 4:**
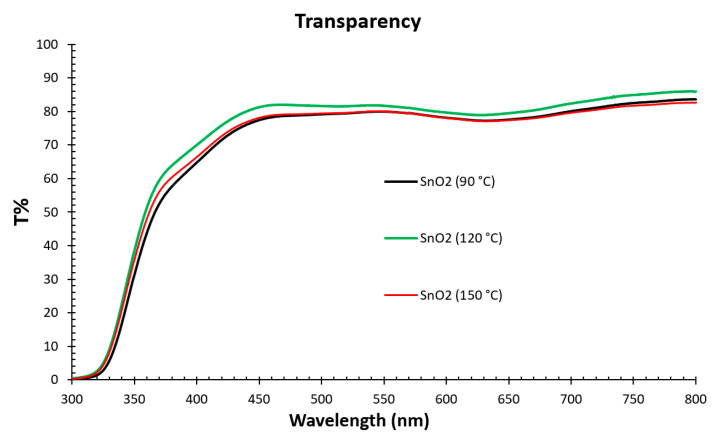
The transparency of SnO_2_ film annealed at 90 °C, 120 °C, and 150 °C as a function of wavelengths.

Figure 4 shows the transparency spectra of the SnO_2_ film annealed at temperatures of 90 °C, 120 °C, and 150 °C. SnO_2_ films deposited on the FTO glass substrate annealed at 120 °C have an average transmittancy of 85% in the visible region and a slight antireflection effect at some wavelength range [21]. The high transparency reduces the optical losses and increases the absorption of light in the perovskite film. The SnO_2_ annealed at 120 °C has around 5% higher transparency for wavelengths between 350 nm and 800 nm compared to other SnO_2_ films annealed at 90 °C and 150 °C.

**Figure 5:**
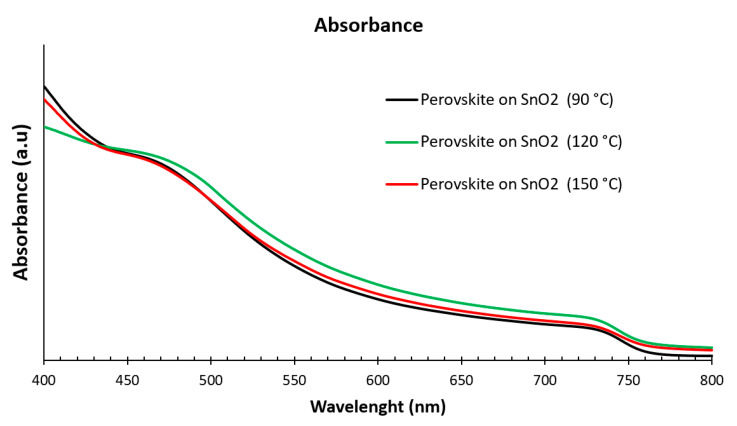
The optical absorption spectra of perovskite films deposited on SnO_2_ film annealed at 90 °C, 120 °C, and 150 °C.

Figure 5 illustrates the optical absorption of perovskite film deposited on SnO_2_ film annealed at 90 °C, 120 °C, and 150 °C. It is clear that the perovskite active layer deposited on SnO_2_ annealed at 120 °C has a higher absorption coefficient than other perovskite films in the wavelength range of 450–775 nm. There is around a 10% improvement in the performance of the perovskite films deposited on SnO_2_ annealed at 120 °C compared with the perovskite active layer deposited on SnO_2_ annealed at 90 °C and 150 °C. The perovskite active layer used in this study, when deposited on SnO_2_ annealed at 120 °C, performed better when tested for light-harvesting devices. This improvement is due to larger grain size, which leads to an increase in the current density and decreased recombination rates when the device is subjected to light tests [27].

**Figure 6:**
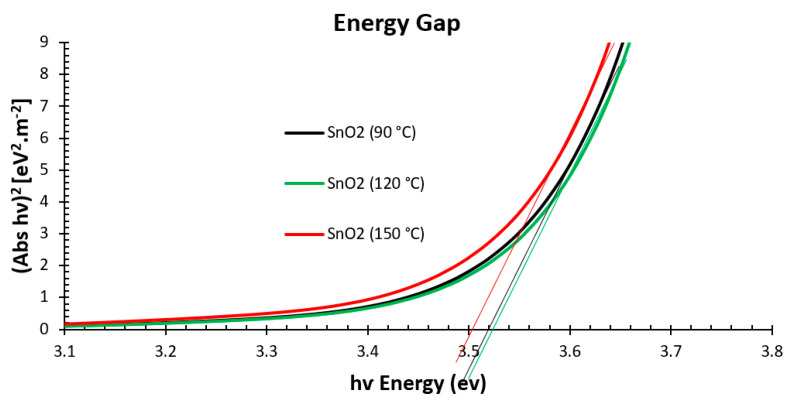
Energy gap extrapolation using Tauc plot of SnO_2_ film annealed at 90 °C, 120 °C, and 150 °C.

Figure 6 displays the absorbance edge of the SnO_2_ annealed with different temperatures. This figure also shows (Abs hυ)^2^ against hυ (in eV) found from the transmission spectra [35]. The E_g_ bandgap of SnO_2_ annealed at 120 °C or 90 °C obtained from the Tauc plot was 3.52 eV, while the E_g_ was 3.5 eV for SnO_2_ annealed at 150 °C. A wider E_g_ offers better hole-blocking ability and can reduce high-energy photon losses, resulting in lower photogenerated carrier losses [36].

**Figure 7:**
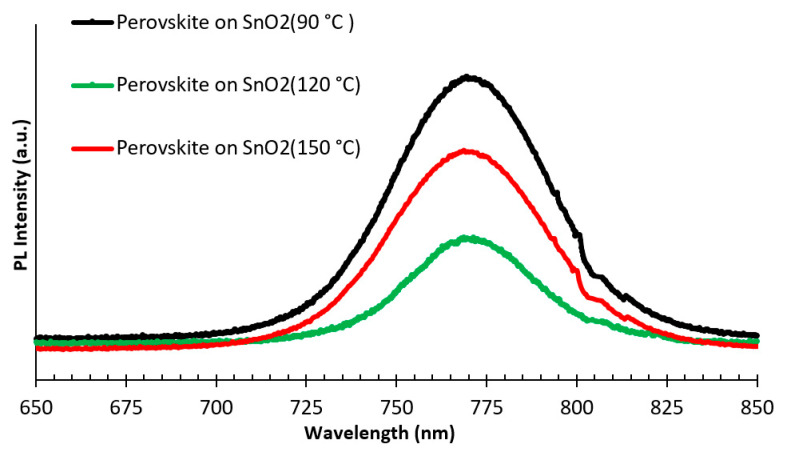
The photoluminescence (PL) spectra of perovskite films deposited on SnO_2_ annealed at 90 °C, 120 °C, and 150 °C.

To study the defect and charge extraction characteristics of perovskite deposited on SnO_2_ annealed at different temperatures, photoluminescence (PL) measurements of these different perovskite films deposited on SnO_2_ were analyzed. Perovskite films deposited on SnO_2_ annealed at 120 °C showed quenching of the perovskite emission peak at 770 nm. Figure 7 displays the PL spectra of perovskite active layers deposited on SnO_2_ annealed at 90 °C, 120 °C, and 150 °C. From the PL spectra of perovskite deposited on SnO_2_ annealed at 90 °C, it can be observed that a much stronger PL intensity peak results from high recombination rates in these films. When applying the perovskite active layer on an SnO_2_ film annealed at 120 °C, the PL spectra peak exhibits notable quenching, indicating effective electron extraction of the ETL and a fast charge transfer. This might be attributed to a comparatively low oxygen vacancy defect density present in the ETL prepared under these conditions [21].

**Figure 8:**
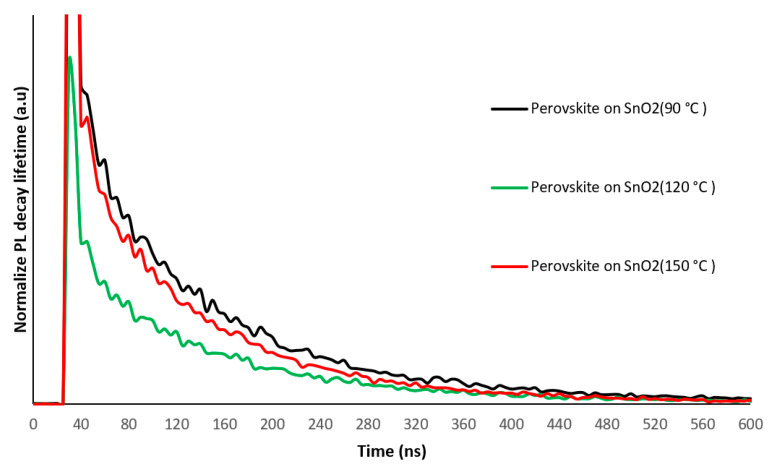
PL decay lifetime of perovskite deposited on SnO_2_ annealed at 90 °C, 120 °C, and 150 °C.

Figure 8 shows the PL decay lifetime of perovskite deposited on SnO_2_ annealed at different temperatures. It can be seen that the PL decay lifetime of perovskite deposited on SnO_2_ annealed at 120 °C was 160 ns, while the lifetimes of perovskite deposited on SnO_2_ annealed at 90 °C or 150 °C were 112 ns and 125.5 ns, respectively. The longer carrier lifetime promotes the extraction and transfer of electrons from the perovskite active layer to the ETL before recombination occurs. This is consistent with the PL results [37].

**Figure 9:**
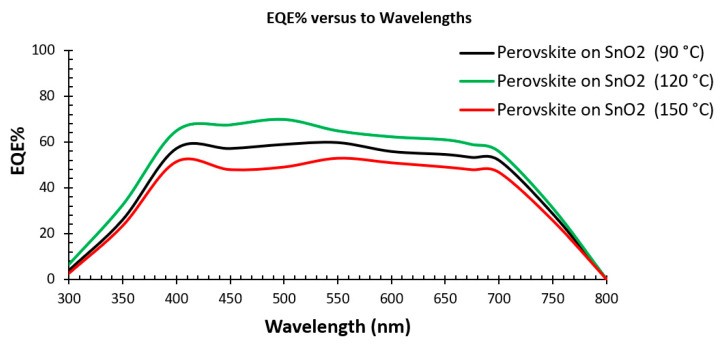
The External Quantum Efficiency (EQE) of the PSCs deposited on SnO_2_ annealed at 90 °C, 120 °C, and 150 °C.

Figure 9 illustrates the external quantum efficiency (EQE) spectra of perovskite deposited on SnO_2_ annealed at 90 °C, 120 °C, and 150 °C. It is found that perovskite deposited on SnO_2_ annealed at 120 °C has larger EQE values at the wavelength range between 300 nm and 800 nm, leading to a higher current density when the device is tested under sunlight conditions. The EQE graphs for perovskite deposited on SnO_2_ annealed at different temperatures exhibited a continued photocurrent generation at wavelengths between 300 and 800 nm and fall outside this wavelength range. The EQE data followed the PL response measurements, indicating that a low PL peak offers larger EQE values [21].

**Figure 10:**
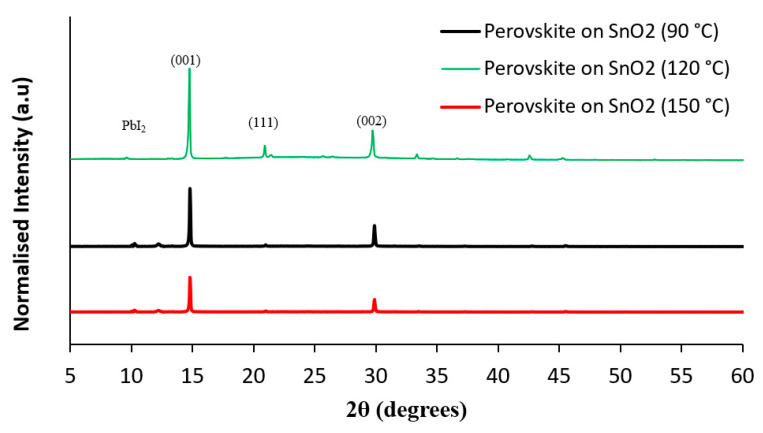
The XRD pattern of the PSCs deposited on SnO_2_ annealed at 90 °C, 120 °C, and 150 °C.

XRD measurements were performed to investigate the effect of changing annealing temperatures of the SnO_2_ films on the surface quality, crystallinity, and phase structure of the perovskite active layer. It can be seen from Figure 10 that the XRD intensity of the (001) plane of the perovskite deposited on SnO_2_ annealed at 120 °C is higher than the perovskite deposited on SnO_2_ annealed at 90 °C and 150 °C. This indicates improved crystallinity of the perovskite active layer with a “high absorption in the short wavelength region” [31]. These results are consistent with the PL spectra results. Our results exhibited a decrease in the PbI_2_ peak intensity for perovskite devices deposited on SnO_2_ annealed at 120 °C compared to other devices deposited on SnO_2_ annealed at 90 °C and 150 °C. This led to a decrease in the hysteresis phenomenon when devices are subjected to current–voltage measurement under sunlight and improved the efficiency of the PSC devices [38].

### 3.2. J-V Characteristics of Perovskite Solar Cells

**Figure 11:**
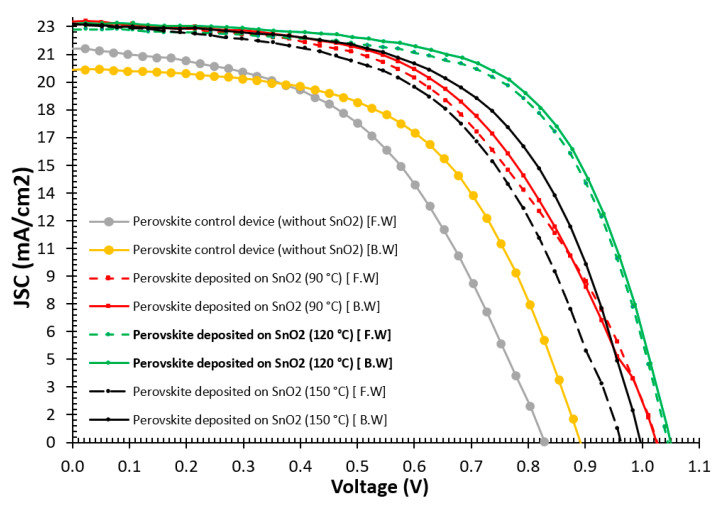
J-V characteristic curves for forward and backward of PSCs devices deposited on SnO_2_ annealed at 90 °C, 120 °C, and 150 °C.

The annealing temperature of SnO_2_ was changed from 90 °C to 150 °C to achieve the optimum annealing temperature that produced the best device efficiency. Device reproducibility is important for PSC performance with the aim of achieving the highest PCE [39]. Ten samples were fabricated using SnO_2_ in each experimental run to ensure reproducibility. Table 1 shows key parameters of the perovskite devices (average of 10 samples) deposited on SnO_2_ at different annealing temperatures. It is demonstrated from the J-V characteristic curves in Figure 11 that perovskite devices deposited on SnO_2_ annealed at 120 °C yielded the highest average efficiency of 15% based on an active area of 0.36 cm^2^. The device efficiency improvements are due to the increased perovskite grain size and the decreased recombination rates in the active perovskite layer. The hysteresis index (H.I) was calculated using Formula (1). The hysteresis phenomenon is caused by “charge accumulation, ion migration, trap-assisted charge recombination, and charge redistribution when the voltage is swept forward and backward [40,41,42]. The average PCE of the control sample without SnO_2_ was 9.4% with an H.I of 14.9%. The drop in efficiency of the control sample is attributed to decreased charge extraction, increased R_s_, and charge recombination [7]. The H.I for perovskite devices deposited on SnO_2_ annealed at 120 °C was less than that used at other annealing temperatures. The perovskite deposited on SnO_2_ annealed at 120 °C produced around 16% higher efficiency than the PSCs deposited on SnO_2_ annealed at 90 °C or 150 °C. It is indicated from Table 1 that the open circuit voltage (V_oc_) of the perovskite deposited on SnO_2_ annealed at 120 °C is 1034 mV, while the average open circuit voltages of other perovskite devices deposited on SnO_2_ annealed at 90 °C and 150 °C were 1026 mV and 982 mV, respectively. The fill factor (FF) of the perovskite deposited on SnO_2_ annealed at 120 °C was 60.8%, while the average FFs of other perovskite devices deposited on SnO_2_ annealed at 90 °C and 150 °C were 51.8% and 55%, respectively. These improvements of perovskite devices deposited on SnO_2_ annealed at 120 °C are attributed to the decrease in the R_s_ from 11.25 to 7.7 Ω·cm^2^. This was also the reason for the increased current density of 22.65 mA/cm^2^.

Generally, perovskite devices fabricated at the ambient laboratory conditions of 25 °C temperature and 45% humidity using SnO_2_ as an ETL, annealed at 120 °C, showed higher PCE and lower hysteresis than other devices using SnO_2_ annealed at 90 °C or 150 °C. These devices also compared well with devices prepared using TiO_2_ ETLs in place of SnO_2_ [14].

Hysteresis index (H.I)% = (EFF%_(B.W)_ − EFF%_(F.W)_)/(EFF%_(B.W)_) (1)

## 4. Conclusions

Tin oxide (SnO_2_) was systematically annealed at various temperatures and explored as an ETL. Our results showed that changing the annealing temperature from 90 °C to 150 °C can affect the power conversion efficiency of the PSCs. The average PCE for 10 samples using SnO_2_ annealed at 120 °C as an ETL was 15% with a 1.3% hysteresis index. The PCE of PSCs fabricated under ambient laboratory conditions with SnO_2_ annealed at 120 °C increased from 12% to 15% based on a 0.36 cm^2^ active device area. It should be noted that an active area of 0.36 cm^2^ is larger than most reported in the literature. The average PCE of the control sample without SnO_2_ was 9.4% with H.I of 14.9%. The electrical, optical, and morphological properties of SnO_2_ layers annealed at various temperatures were investigated and correlated to the PSC device performance in terms of efficiency. The perovskite layer deposited on SnO_2_ annealed at 120 °C has shown an increase in grain size to 180nm and a reduction in the R_s_ from 11.25 Ω·cm^2^ to 7.7 Ω·cm^2^. There is around 10% enhancement in the absorption coefficient of the perovskite film deposited on SnO_2_ annealed at 120 °C compared to the annealed temperatures of 90 °C and 150 °C. The reduction in R_s_ has also improved the FF of the perovskite devices to 60.8%. It was found that the PSCs deposited on SnO_2_ annealed at 120 °C achieved the highest efficiency due to their 85% transparency, minimal surface roughness RMS of 14 nm, low contact angle of 4.1°, PL decay lifetime of 160 ns, and “lower PL intensity, indicating faster charge extraction before recombination occurs”. This study demonstrates the feasibility of low-temperature-processed perovskite devices, paving the way towards low-cost fabrication techniques that are compatible with flexible substrates.

## Figures and Tables

**Table 1:** Key parameters of PSCs devices deposited on SnO_2_ annealed at 90 °C, 120 °C, and 150 °C. The active area used in this study was 0.36 cm^2^.

Sample Description	Sweep Direction	EFF%	HI%	FF%	Voc [mV]	Jsc [mA/cm^2^]	Vmax [mV]	Jmax [mA/cm^2^]	Isc [mA]	R_shunt_ [Ω·cm^2^]	R_series_ [Ω·cm^2^]
Perovskite control device without SnO_2_	F.W	8.72		44.5	829	21.3	526	16.6	7.6	865	15
	B.W	10.25	14.9	50.4	891	20.2	626	16.2	7.3	1250	13.4
	Avg.	9.4		47.4	860	20.7	576	16.4	7.4	1057	14.2
Perovskite deposited on SnO_2_ (90 °C)	F.W	12.2		51	1025	22.48	654	18.5	8.1	687	11.7
	B.W	12.8	4.6	52.7	1027	22.5	680	18.4	8.2	6863	10.8
	Avg.	12.5		51.85	1026	22.49	667	18.45	8.15	3775	11.25
Perovskite deposited on SnO_2_ (120 °C)	F.W	14.9		59.8	1023	22.7	749	19.16	7.9	7532	7.65
	B.W	15.1	1.3	61.8	1045	22.6	765	19.5	8.13	7698	7.7
	Avg.	15		60.8	1034	22.65	757	19.35	8.02	7615	7.7
Perovskite deposited on SnO_2_ (150 °C)	F.W	12.05		53	963	22.45	655	17.98	8.08	412	9.2
	B.W	13.4	7.9	57	1001	22.5	710	18.5	8.1	936	8.4
	Avg.	12.7		55	982	22.45	682.5	18.2	8.1	674	8.8

## Data Availability

The original contributions presented in the study are included in the article, further inquiries can be directed to the corresponding author.
